# Local delivery of hydrogel encapsulated vascular endothelial growth factor for the prevention of medication-related osteonecrosis of the jaw

**DOI:** 10.1038/s41598-021-02637-w

**Published:** 2021-12-03

**Authors:** Dileep Sharma, Stephen Hamlet, Cedryck Vaquette, Eugen Bogdan Petcu, Poornima Ramamurthy, Saso Ivanovski

**Affiliations:** 1 College of Medicine and Dentistry, James Cook University, Cairns Campus, PO Box 6811, Cairns, 4870 Australia; 2 Australian Institute of Tropical Health and Medicine, James Cook University, Cairns, Australia; 3 Menzies Health Institute Queensland, School of Medicine and Dentistry, Griffith University, Gold Coast Campus, Gold Coast, 4222 Australia; 4 School of Dentistry, Faculty of Health and Behavioral Sciences, The University of Queensland, Herston Campus, Brisbane, 4006 Australia; 5 New York Institute of Technology College of Osteopathic Medicine (NYIT), Old Westbury, NY 11545 USA; 6 School of Dentistry and Oral Health, Griffith University, Gold Coast, Australia

**Keywords:** Dental diseases, Experimental models of disease

## Abstract

The anti-angiogenic effects of bisphosphonates have been hypothesized as one of the major etiologic factors in the development of medication-related osteonecrosis of the jaw (MRONJ), a severe debilitating condition with limited treatment options. This study evaluated the potential of a gelatine-hyaluronic acid hydrogel loaded with the angiogenic growth factor, vascular endothelial growth factor (VEGF), as a local delivery system to aid in maintaining vascularization in a bisphosphonate-treated (Zoledronic Acid) rodent maxillary extraction defect. Healing was assessed four weeks after implantation of the VEGF-hydrogel into extraction sockets. Gross examination and histological assessment showed that total osteonecrosis and inflammatory infiltrate was significantly reduced in the presence of VEGF. Also, total vascularity and specifically neovascularization, was significantly improved in animals that received VEGF hydrogel. Gene expression of vascular, inflammatory and bone specific markers within the defect area were also significantly altered in the presence of VEGF. Furthermore, plasma cytokine levels were assessed to determine the systemic effect of locally delivered VEGF and showed similar outcomes. In conclusion, the use of locally delivered VEGF within healing extraction sockets assists bone healing and prevents MRONJ via a pro-angiogenic and immunomodulatory mechanism.

## Introduction

Bisphosphonates (BPs), stable analogues of pyrophosphates, are chelating agents with a high binding affinity for bone mineral that accumulate in bone and are subsequently released during bone resorption^1^. BPs are subsequently reincorporated into newly formed bone or internalized by osteoclasts where they inhibit enzymes in the mevalonate pathway causing osteoclasts to lose their normal function^2,3^. To inhibit osteoclast function, BP therapy has proven to be one of the most efficient ways to treat benign and malignant diseases characterized by high bone turnover, such as metastatic bone diseases (breast cancer, prostate cancer and multiple myeloma primary sites), osteoporosis, Paget’s disease and pediatric osteogenesis imperfecta^4–6^.

Notwithstanding these therapeutic effects, BP use has also been associated with significant adverse effects that occur selectively in jaw bones often but not always, following tooth extraction and surgical procedures. Bisphosphonate-Related Osteonecrosis of the Jaw (BRONJ), now termed as Medication-related Osteonecrosis of the Jaw (MRONJ), results from the direct effects of BPs on bone cells (osteoblasts, osteoclasts and osteocytes) and vascular tissue which ultimately leads to a necrotic lesion^7–9^. BPs have been shown to impede vascularity and angiogenesis not only through their direct effect on blood vessels, endothelial cells and their precursors (in situ and in circulation), but also by inhibition of endothelial differentiation from multipotent stem cells^10–17^. The anti-angiogenic action of Zoledronic Acid (ZA), a highly potent intravenously used BP, has been shown to significantly affect soft tissue and osseous healing following dental extraction^18,19^. Furthermore, genetic polymorphisms within VEGF genes have also been associated with increased risk of MRONJ^20^. These findings provide a sound biological rationale for the clinical application of a local angiogenic agent such as vascular endothelial growth factor (VEGF), to counter the local anti-angiogenic effect of BPs, ultimately resulting in an enhanced quality and rate of tissue healing.

Vascular endothelial growth factor (VEGF), a potent angiogenic growth factor plays a critical role in initial phase of the wound healing cascade. In vitro, VEGF has been shown to regulate osteoblastic differentiation, chemotactic migration and survival through an autocrine feedback mechanism^21,22^. Furthermore, direct association between local angiogenesis and bone growth during the formative stages of bone development, callous formation during fracture repair and distraction osteogenesis have all been well documented^23–25^.

Hydrogels, based on their ability to incorporate growth factors that are predictably released over a sustained period in a localized fashion, are a suitable delivery vehicle for growth factors such as VEGF. Specifically, the gelatine-hyaluronic acid (Gel-HA) [α-1,4-d-glucuronic acid-β-1,3-*N*-acetyl-d-glucosamine]_n_, based gel system has a proven potential as a delivery vehicle for various bioactive molecules^26–28^. HA is a naturally occurring, hydrophilic, non-immunogenic glycosaminoglycan and is known to play a significant role during morphogenesis and wound healing^29,30^. HA has been shown to enhance osseous healing when combined with osteo-conductive materials and bone substitutes, including collagen, calcium phosphate, demineralized freeze-dried bone allografts and autologous bone grafts^31,32^.

Whilst previous studies have evaluated the effect of locally delivered VEGF in various bone regenerative procedures, and although there is a sound rationale for the use of this growth factor to counter the anti-angiogenic effects of BPs, no studies have evaluated its application as a preventive approach in developing MRONJ lesions. Hence, the aim of this study was to evaluate the effects of locally delivered, sustained release of VEGF using Gel-HA-based hydrogels, on the prevention of MRONJ in a rodent model.

## Results

### In vitro VEGF-gel release kinetics

**Figure 1:**
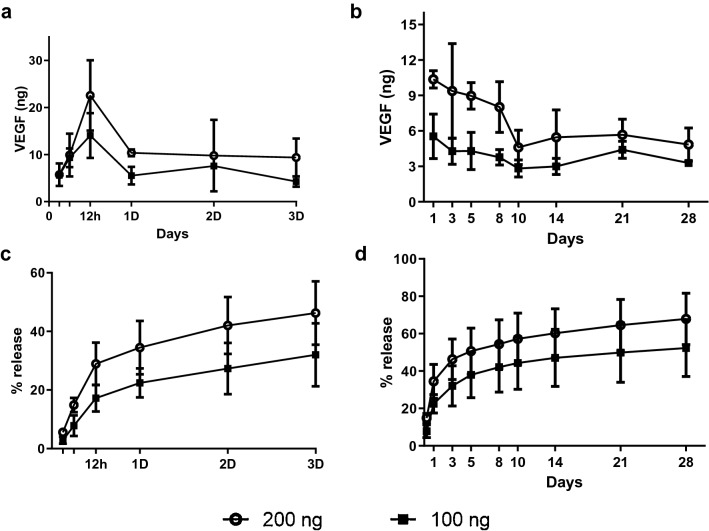
Release profile of VEGF from the HA hydrogel. Short term (**a**,**c**) and long term (**b**,**d**) release kinetics show an initial burst release followed by a sustained release profile.

The in vitro VEGF release profile quantified by ELISA showed similar release kinetics for the two different doses tested and was proportional to the initial loading dosage (Fig. 1). There was a rapid release (18–28%) of total VEGF that peaked at 12 h in both the 100 and 200 ng VEGF-loaded gels. By the end of 3 days, 25–30% of the VEGF had been released from the 100 ng gel and 38–42% from the 200 ng gel. After a week, the rate of release was reduced but was sustained between 4 and 8 ng per week over the rest of the observation period of 28 days. A total of 108.3 ± 25.27 ng of VEGF was released from the 200 ng gel and 67.87 ± 9.12 ng from the 100 ng loaded gel by the end of 28 days, demonstrating that only partial release of the growth factor was achieved during the experimental period. As the 200 ng dose could rapidly deliver 20 to 30 ng of VEGF within twelve hours and sustained release thereafter, it was deemed to have the greater potential for inducing in vivo neovascularization. Hence, the 200 ng dose was utilised in the rest of the study.

### Gross morphological examination

**Figure 2:**
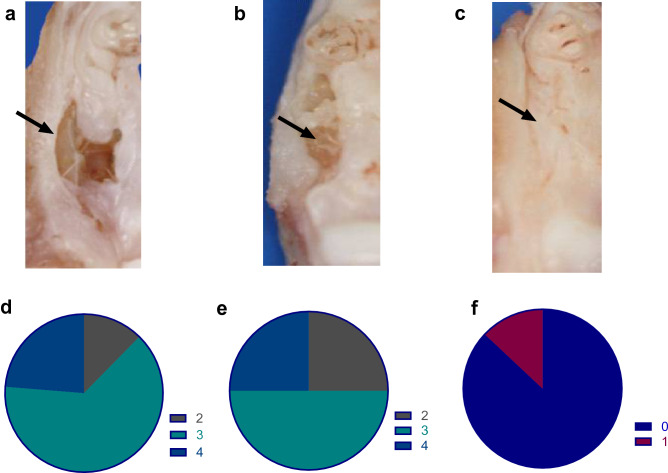
Clinical images of MRONJ lesion/defect area (black arrows) in no-gel (**a**), gel-alone (**b**) and VEGF-gel groups (**c**); Pie-charts demonstrating the distribution of MRONJ classification stages in the three treatment groups; no-gel (**d**), gel-alone (**e**) and VEGF-gel group (**f**).

The maxillae were dissected carefully without disturbing the overlying mucosa. Gross osteonecrosis, in the form of incomplete wound closure, inflammation and presence of necrotic bone (sequestra) was evident in the maxillary defects with no-gel group, and to a lesser extent, in the gel-alone group, after 4 weeks healing of the extraction defect site (Fig. 2a–c). However, complete wound closure was noted in the rats treated with VEGF hydrogel. MRONJ grading in the no gel group revealed that 62% of the rats were classified as grade 3 and 23% as grade 4 with the remainder being grade 2. In the gel-alone group, 50% were classified as grade 3 MRONJ and the rest were equally split between grade 2 and grade 4. However, the VEGF-gel group demonstrated drastically improved healing, with the majority of animals recording grade 0 (87%) with the rest (13%) being rated grade 1 (Fig. 2d–f).

### Histological findings

#### Quantification of osteonecrosis

**Figure 3:**
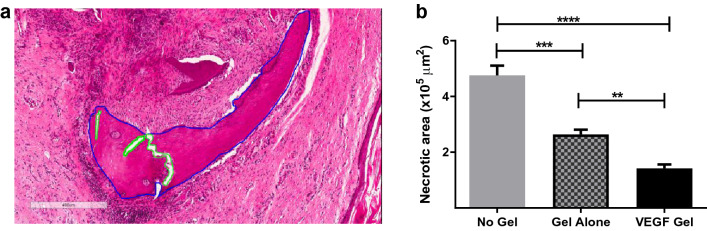
Representative photomicrographs of annotated necrotic area (defect site) with empty lacunae from no-gel group (**a**); quantification of necrotic area (**b**) showed a significant positive effect of the VEGF-gel compared to no-gel and gel-alone groups (****p < 0.0001, ***p < 0.001, **p < 0.01). Blue outlines indicate included areas and green outlines depict excluded areas whilst quantification was performed.

Quantification of osteonecrosis in the defect site showed significantly higher areas of necrotic bone (empty lacunae) in the untreated (no-gel) animals at the end of 4 weeks healing (Fig. 3a,b). These untreated animals showed a significantly higher necrotic area compared to the VEGF treated group (4.78 ± 0.35 × 10^5^ µm^2^ vs 1.42 ± 0.14 × 10^5^ µm^2^, p < 0.0001; Fig. 3b). Further, the gel-alone group also had significantly lower areas of necrotic bone compared to the untreated group (2.64 ± 0.17 × 10^5^ µm^2^, p = 0.0001) but significantly higher osteonecrosis than the VEGF treated group (p = 0.009). These results suggest a positive role for VEGF in the prevention of ZA induced MRONJ (Fig. 3b).

#### Quantification of inflammatory infiltrate

**Figure 4:**
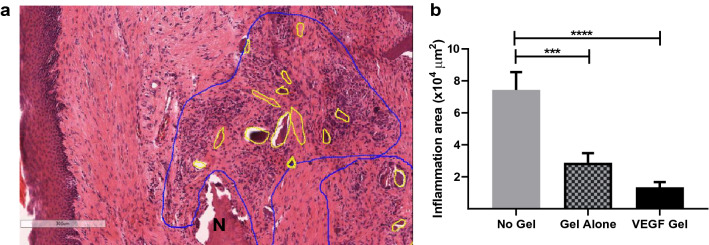
Representative photomicrograph of annotated inflammatory infiltrate around the necrotic (N) area in the defect site from no-gel group (**a**). Quantification (**b**) showed a significant reduction of inflammatory infiltrate in the VEGF-gel compared to no-gel and gel-alone groups (****p < 0.0001, ***p < 0.001).

Significantly larger areas of inflammatory reaction were noted within the extraction sockets and around the sequestrum in the untreated group, in comparison to the VEGF treated group (7.01 ± 0.93 × 10^4^ µm^2^ vs 1.29 ± 0.18 × 10^4^ µm^2^, p < 0.0001). The gel-alone group showed marginally higher volume of infiltrate (2.92 ± 0.24 × 10^4^ µm^2^) than the VEGF group (p = 0.142) suggesting that VEGF had minimal role in reducing the inflammatory component. However, the inflammatory infiltrate in the no-gel and gel-alone groups differed significantly (p = 0.0007; Fig. 4) suggesting that HA-gel alone can significantly reduce the inflammatory component.

#### Quantification of vascularity

**Figure 5:**
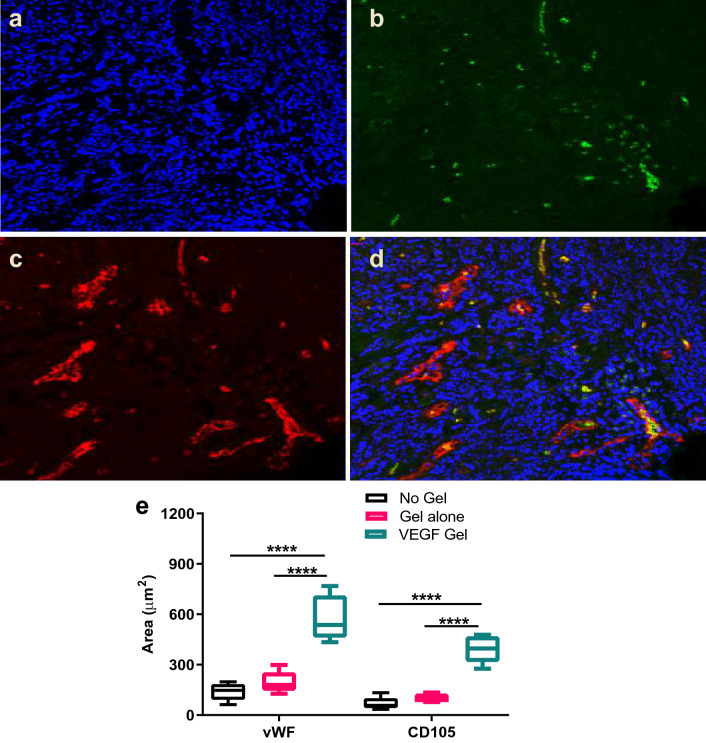
Representative image from VEGF gel group with double immunofluorescence staining for vascular markers. Nuclear stain DAPI (blue, **a**), CD105 (green, **b**), vWF (red, **c**), and merged photomicrograph (**d**). Quantification of staining (**e**) showed significant difference between VEGF-gel and the other groups in vWF and CD105 expression (****p < 0.0001).

vWF staining was significantly higher in the VEGF-gel (577 ± 60 µm^2^) compared to the no-gel group (140 ± 23 µm^2^, p < 0.0001) suggesting a positive role of the VEGF-gel on total vascularity (Fig. 5). The gel-alone group also showed significantly lower vWF staining in comparison to the VEGF-gel group (196 ± 29 µm^2^, p < 0.0001) confirming the role of VEGF in increasing vascular density. The difference between the no-gel and gel-alone group did not differ significantly (p = 0.2495).

Similar trends were observed with CD105 expression within the healing sockets. The stained areas were the highest in the VEGF-gel group (393 ± 36 µm^2^) followed by the gel-alone (103 ± 11 µm^2^) and no-gel groups (69 ± 17 µm^2^). VEGF-gel was seen to significantly increase CD105 expression compared to both the no-gel (p < 0.0001) and gel-alone (p < 0.0001) groups. Again, the difference between the no-gel and gel-alone groups did not reach a statistically significant level (p = 0.4805), suggesting that VEGF played an important role in neovascularization.

### Gene expression

#### Gingival tissue

**Figure 6:**
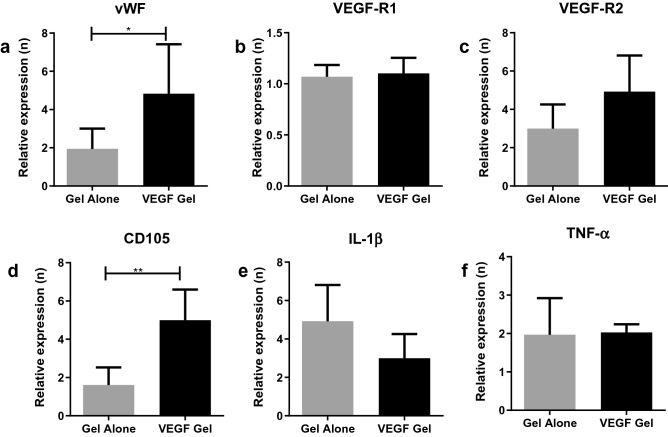
Relative gene expression within the peri-lesion (defect) soft tissue normalized to no-gel levels; vWF (**a**), VEGF-R1 (**b**), VEGF-R2 (**c**), CD105 (**d**), IL-1β (**e**), and TNF-α (**f**) (**p < 0.01, *p < 0.05).

Expression of the vascular markers vWF, VEGF-R1, VEGF-R2 and CD105 along with the inflammatory cytokines IL-1β and TNF-α was normalized to that observed in the no-gel group (Fig. 6). vWF gene expression (Fig. 6a) was shown to be two to eightfold higher in the VEGF-gel and two to threefold higher in the gel-alone groups compared to the no-gel group, with the difference between VEGF-gel and gel-alone being statistically significant (p = 0.04). In contrast, VEGF-R1 showed a minimal increase in expression in the VEGF-gel and gel-alone groups with no significant difference between these groups suggesting that the endogenous soft tissue expression of VEGF-R1 seems to be unaffected by the VEGF (Fig. 6b). VEGF-R2 expression (Fig. 6c) showed a five to sevenfold increase in the VEGF-gel and a three to fivefold increase in the gel-alone groups compared to the no-gel group although no statistical difference was reached when comparing VEGF-gel to gel-alone. The increased expression of CD105 (Fig. 6d) in the presence of VEGF-gel (fivefold) was significantly higher than that associated with the gel-alone group (twofold) (p = 0.0022).

Although gene expression of the inflammatory marker IL-1β (Fig. 6e) showed an increase in both the VEGF and gel-alone groups compared to the no-gel group, the expression in the VEGF-gel group was lower than the gel-alone group (two to threefold c.f. five to sevenfold). In the case of TNF-α gene expression, an increase two to threefold higher was noted in both the VEGF-gel and gel-alone groups (Fig. 6f). It was noteworthy that there was no statistically significant difference between the VEGF-gel and gel-alone group for both the inflammatory cytokines tested, suggesting that the addition of VEGF to the gel did not trigger a major inflammatory process in the soft tissues around the MRONJ lesion.

#### Bone tissue

**Figure 7:**
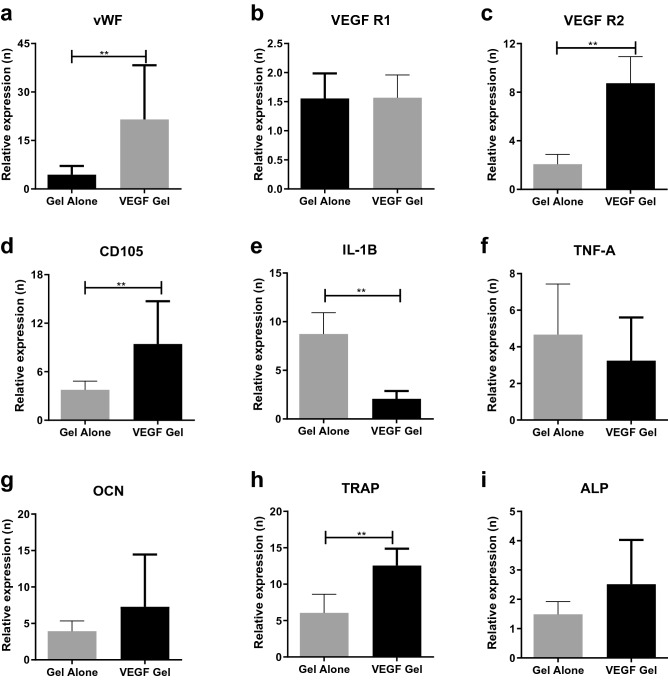
Relative gene expression within MRONJ lesion (defect) hard tissue normalized to no-gel levels; vWF (**a**), VEGF-R1 (**b**), VEGF-R2 (**c**), CD105 (**d**), IL-1β (**e**), TNF-α (**f**), OCN (**g**), TRAP (**h**) and ALP (**i**) **p < 0.01.

Gene expression in the bone tissue (Fig. 7) was also quantified to evaluate the effect of VEGF-gel on vascular (vWF, VEGF-R1, VEGF-R2, CD105), inflammatory (IL-1β and TNF-α) and bone specific markers (OCN, TRAP and ALP). An increase in vWF expression (Fig. 7a) was shown to be significantly higher in the VEGF-gel group (30–40 fold) when compared to a five to sevenfold increase in the gel-alone group (p = 0.0065). However, similar to the soft tissue pattern of expression, VEGF-R1expression (Fig. 7b) in the hard tissue was only around 1.5-fold greater in both the VEGF-gel and gel-alone groups suggesting that VEGF did not significantly affect the expression of this endogenous marker. In contrast, the increased expression of VEGF-R2 (Fig. 7c) was significantly higher in the VEGF-gel group (tenfold) compared to a two to threefold increase in the gel-alone group (p = 0.0022). The gene expression of CD105 (Fig. 7d) was increased up to tenfold in the presence of VEGF-gel compared to a fourfold increase in the gel-alone group, and this difference was statistically significant (p = 0.0022).

IL-1β levels (Fig. 7e) in the VEGF-gel group showed significantly lower expression levels (twofold) compared to the negative control no-gel group, while there was a tenfold increase in the gel-alone group (p = 0.0022). TNF-α gene expression (Fig. 7f) also showed similar trends, although the difference between the groups did not reach statistical significance (p = 0.3052).

TRAP gene expression levels (Fig. 7h) were also elevated in the VEGF-gel group compared to gel-alone group (15-fold vs eightfold, p = 0.0043). There was also a general trend for increased OCN and ALP gene expression (Fig. 7g) but the difference between the VEGF-gel and gel-alone groups was not statistically significant (Fig. 7i).

### Peripheral cytokine levels

**Figure 8:**
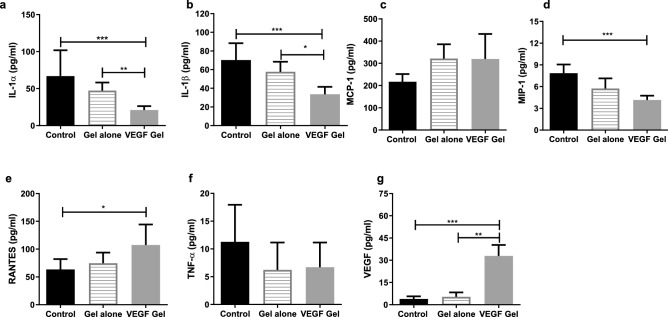
Plasma cytokine levels showed a statistically significant modulation in the VEGF-gel group. IL-1α (**a**), IL-1β (**b**), MCP-1 (**c**), MIP-1 (**d**), RANTES (**e)**, TNF-α (**f**) and VEGF (**g**) (***p < 0.001, **p < 0.01, *p < 0.05).

A multiplex ELISA was performed to evaluate the effect of the locally delivered VEGF on peripheral cytokine levels (Fig. 8). Peripheral IL1-α levels (Fig. 8a) were significantly reduced in both the VEGF-gel group (21 ± 5.33 pg mL^−1^) and to a lesser extent in the gel-alone group (47.34 ± 10.88 pg mL^−1^) when compared to the no-gel group (66.92 ± 12.35 pg mL^−1^; p = 0.0005 and p = 0.0071, respectively). A similar trend was observed in IL-1β levels (Fig. 8b) which were reduced in both the VEGF-gel group (33.63 ± 2.78 pg mL^−1^) and to a lesser extent in the gel-alone group (57.66 ± 3.82 pg mL^−1^) when compared to the no-gel group (70.29 ± 6.38 pg mL^−1^; p = 0.0002 and p = 0.0194 respectively).

Plasma TNF-α levels also followed the same pattern but none of the inter-group differences reached statistically significant levels (Fig. 8c). MCP-1 levels were lower in both the gel-alone and no-gel groups but higher in the VEGF-gel group. (Fig. 8d) MIP-1 levels (Fig. 8e) were significantly elevated in the VEGF-gel group compared to the no-gel group (7.861 ± 0.421 pg mL^−1^ vs 4.161 ± 0.201 pg mL^−1^, p = 0.0002). RANTES levels (Fig. 8f) also showed a significant elevation in the VEGF-gel group (107.5 ± 13.05 pg mL^−1^) compared to the no-gel group (63.43 ± 6.58 pg mL^−1^, p = 0.0101).

The largest effect of the VEGF-gel was evident on peripheral VEGF levels in the rats (Fig. 8g). Mean VEGF levels significantly increased more than tenfold in the presence of VEGF-gel in comparison to the no-gel (32.88 ± 2.636 pg mL^−1^ vs 3.868 ± 0.6298 pg mL^−1^ p = 0.0007) and gel-alone groups (5.363 ± 1.035 pg mL^−1^ p = 0.0056).

## Discussion

Management of established MRONJ lesions is a significant clinical challenge, and unpredictable or sub-optimal outcomes have been obtained using a range of surgical and conservative treatment modalities^9,33–35^. Hence, a preventive approach aimed at addressing the most-likely aetio-pathogenic causes of the disease is a sound management strategy. In addition to affecting a variety of biological functions, ZA (and most BPs to some extent) can significantly affect local vascularity and angiogenesis through their effect on the circulatory system and specifically by the inhibition of endothelial differentiation from multipotent stem cells^10–17^. Therefore, a novel approach to prevent the occurrence of this condition by locally replenishing the suppressed angiogenic stimuli, could be a viable option for the management of susceptible patients undergoing BP treatment. In this study, we have shown that the local delivery of VEGF via a gelatine-hyaluronic acid hydrogel significantly reduced the development of MRONJ in an rat model of the disease.

VEGF is a 45 kDa homodimeric glycosylated protein with at least twelve alternatively spliced variants, some with pro-angiogenic and some with anti-angiogenic properties^36^. VEGF_121_, VEGF_145_, VEGF_165_, VEGF_189_, and VEGF_206_ have been classically described as pro-angiogenic and differences in their heparin and heparan-sulfate binding ability distinguishes these VEGF isoforms. VEGF_165_ was chosen for local delivery in this study because it is a secreted isoform that can bind to heparin through a unique 44 amino acid-long peptide. Exogenous VEGF has been shown to promote bone repair^37^, and local delivery of VEGF for enhanced bone formation has been achieved using various delivery systems, including calcium-induced alginate hydrogels^37^, and mineralized poly (lactide-co-glycolide) (PLGA) scaffolds^38^.

Hydrogel containing heparin-bound VEGF presents a convenient clinically translatable strategy that has not been investigated as a local delivery vehicle for the management of MRONJ. The HyStem-HP hydrogel used to deliver the VEGF in this study contains a combination of heparin thiol-modified hyaluronan, a thiol-modified denatured collagen (Gelin-S), and a thiol-reactive cross linker, PEGDA (Extralink). The immobilized heparin in the HyStem-HP hydrogel mimics the heparin sulphate proteoglycans normally present in the extracellular matrix. The addition of heparin is an attractive strategy for sustained growth factor delivery as it forms an ionic bond with proteins (in this case VEGF), thus protecting them from proteolysis and resulting in a slower and more sustained release from the hydrogel. Hyaluronic acid (HA) based hydrogels containing heparin have been shown to have a positive role in post-surgical healing following soft tissue grafts, in addition to significantly enhancing osseous healing when used alone or in combination with bone substitutes^31,32,39–46^. HA has also demonstrated anti-inflammatory properties that would be beneficial in MRONJ which is typically associated with an increased inflammatory infiltrate^5,47–49^. Another study by Brierly et al. with similar approach, employing starPEG-RGD-heparin hydrogel, also reported that extraction sites with hydrogel alone treatment had a significantly better healing and reduced MRONJ, in comparison to no gel control sites as noted on microCT and histological analysis^50^. In our study, the HA gel alone showed a significant reduction in necrotic tissue that could be attributed to the pro-healing properties of HA gel. Although HA gel was found to significantly decrease bone necrosis, its anti-inflammatory affect did not reach statistical significance. Improved sealing of the extraction socket upon implantation of the gel resulted in decreased exposure to the oral microenvironment, thus reducing bacterial contamination and subsequent inflammation that may also have enabled some partial healing. However, the addition of VEGF to the HA gel achieved a significant reduction in the inflammatory infiltrate as quantified on histological sections. This finding suggests that in this MRONJ model, the gelatine-HA hydrogel alone was only partially effective in reducing inflammation within the developing lesion, with VEGF being necessary to achieve optimal results.

Neovascularization is an integral part of tissue healing and CD105 is considered an important marker of this process^51^. CD105 (endoglin), a surface structure motif of the transforming growth factor β receptor III (TGFβR-III), is expressed predominantly in endothelial cells^52^. CD105 is known to be highly expressed by proliferating endothelial cells during angiogenesis, and its increase has been observed in blood vessels as early as 2 days and up to 28 days, during post-surgical wound healing^53,54^. Previously, MRONJ-associated mucoperiosteal tissue was reported to show significantly fewer CD105-positive vessels than control samples^55^. In the present study, immunofluorescence staining and quantification of vWF for vascularity and CD105 for new blood vessel formation, showed significant improvement in total vascularity and neovascularization in the VEGF group compared to the no-gel group. This suggests that, in addition to total vascularity, neovascularization is also significantly suppressed by ZA, which in turn is detrimental for early bone healing.

Gene expression analysis of vascular markers (vWF and CD105) in both the soft and hard tissues showed further evidence of the positive angiogenic effects of locally delivered VEGF in this model of MRONJ. Although VEGF-R1 levels (hard and soft tissue) did not seem to be affected, VEGF-R2 was up-regulated in both tissues. This is noteworthy, because although the affinity of VEGF is higher for VEGF-R1, the angiogenic effect of VEGF occurs predominantly through activation of VEGF-R2^56,57^. Furthermore, VEGFR-2 is also known to stimulate secretion of vWF from endothelial cells^58^, thus contributing to the higher immunofluorescent staining noted in our study.

Bone marker gene expression analysis was also performed to evaluate the molecular aspects of the effect of VEGF on bone healing in developing MRONJ lesion. The bone formation markers OCN and ALP were elevated in the VEGF-gel treated animals compared to both the gel-alone and no-gel groups, suggesting that VEGF may positively affect bone formation. However, the differences observed did not reach statistical significance. This finding is supported by a previous study showing that ZA suppresses OCN levels in a rat MRONJ model, which could be counteracted by local application of autologous Platelet-Rich Plasma^59^. Furthermore, TRAP, an osteoclast activity marker known to be suppressed in MRONJ lesions^60,61^, showed a significant increase in gene expression after VEGF delivery (p = 0.0043). Similarly, when the expression of inflammatory genes was assessed, VEGF treatment resulted in much lower expression of IL-1β in comparison to gel-alone treated animals in the soft tissues. A more pronounced and significant reduction in the expression of IL-1β in the VEGF compared to the gel-alone group was noted in the hard tissues. These findings suggest a local immunomodulatory effect of VEGF-gel within the hard and soft tissue compartment of the healing extraction socket.

Low peripheral VEGF levels have been previously associated with MRONJ^43,62^. In human studies, serum VEGF and TGF-β levels were reported to be significantly suppressed after administration of intravenous bisphosphonate, suggesting that peripheral VEGF and inflammatory marker levels could represent a possible predictive marker for susceptibility to MRONJ^43,63^. In our study, we found that locally delivered VEGF significantly increased circulatory VEGF levels, suggesting an additional systemic effect. This appears to elicit a systemic cytokine response which can be attributed to the known autocrine and paracrine effects of VEGF (essential for vascular homeostasis)^64^. Our findings also showed that elevated circulatory levels of inflammatory cytokines 1L-1α and IL-β, a common feature in inflammatory and bone-resorptive conditions^65^, were significantly reduced in the presence of VEGF, suggesting an immunomodulatory systemic response. Although, elevated levels of TNF-α have been associated with MRONJ in human studies, there have been no studies evaluating the peripheral TNF-α levels in a MRONJ model^66^.

Monocyte chemoattractant protein-1 (MCP-1/CCL2) is a chemokine known to have an important role in monocyte-macrophage recruitment, differentiation of osteoclast precursors and chemo-attraction of osteoclasts^67^. MCP-1 is known to be expressed by osteoblasts and acts directly on osteoclast precursors to trigger the process of bone resorption through osteoclastogenesis in the presence of RANK ligand (RANKL)^68^. Recently, an in vitro study reported that ZA administration could significantly reduce production of MCP-1 in osteosarcoma cell lines, thus reducing tumor-induced bone destruction and tumor growth^69^. Pre-treatment of a macrophage-like cell line (J774.1) with the BP alendronate was shown to decrease the production of MCP-1 and macrophage inflammatory protein-1alpha (MIP-1α) in vitro, suggesting that long-term use of BPs might inhibit normal activation and migration of osteoclasts and ultimately cause osteonecrosis^70^. In this study circulatory MCP-1 and more so MIP-1 levels, were elevated in the VEGF group when compared to the gel-alone and no-gel groups, suggesting that a restoration of osteoclastic differentiation and function may lead to improved healing outcomes.

The results of this study showed that the development of MRONJ in a rat model can be prevented by the pro-angiogenic action of locally delivered VEGF via a heparin-hyaluronic acid hydrogel carrier. This was supported by in vivo evidence of enhanced bone healing, histological evidence of reduced osteonecrosis and increased microvessel formation. The up-regulation of pro-angiogenic genes (vWF and VEGF-R2 and CD105) as a result of the VEGF-gel, paralleled a significant increases in systemic VEGF levels. This approach could prove to be a viable and clinically translatable strategy for the prevention of MRONJ lesions in patients on bisphosphonates.

## Materials and methods

### Preparation of VEGF hydrogel delivery system

A chemically defined hydrogel (HyStem-HP, Advanced BioMatrix, Inc, USA) was utilized for the study. Briefly, 1 mL of deionized water (DG) water was transferred aseptically to the HyStem-HP and Gelin-S vials using a syringe and needle to dissolve the lyophilized solids. Both vials were placed horizontally on a tube roller for around 30 min for the solids to fully dissolve resulting in a clear and slightly viscous solution. Hydrogels were prepared by mixing HyStem-HP with Gelin-S in a 1:1 volume ratio and cross-linking this mixture with 2% wv^−1^ polyethylene glycol diacrylate (Extralink) in a 4:1 volume ratio.

To prepare the VEGF-gel, equal volumes of HyStem-HP and Gelin-S were mixed in a 48 well tissue culture plate and VEGF (100 or 200 ng; Recombinant Rat VEGF_165,_ BioVision Incorporated, Milpitas, CA, USA) was added to each well, resulting in a final volume of 0.5 mL of gel, including the 125 µL of Extralink to form the hydrogel.

### In vitro VEGF release kinetics

The VEGF release kinetics from the hydrogel were assessed for each concentration of VEGF in triplicate. Two concentrations (100 ng and 200 ng) hydrogels were incubated (in triplicates) in a solution containing 1 mL phosphate buffered saline (PBS) supplemented with Bovine serum albumin (BSA 1%), heparin (10 µg mL^−1^) and EDTA (1 mM at 37 °C) on an orbital shaker at 150 rpm. At various time points from 3 h to 28 days, 1.0 mL of the solution was collected for VEGF release analysis. Fresh incubation solution (1 mL) was added at each collection time-point to maintain a constant volume. All the samples were stored at -80 °C prior to analysis.

A rat VEGF enzyme-linked immunosorbent assay (Biosensis, Sapphire Bioscience, NSW, Australia) was used to quantitate the release profile of VEGF from the hydrogel according to the manufacturer’s instructions. Briefly, reconstituted VEGF standard solution and samples (in triplicate) were incubated at 37 °C for 90 min in anti-VEGF pre-coated plates. Subsequently, after appropriate washing, a secondary biotinylated antibody was added to the wells of the plate that was incubated at room temperature for further 60 min. Color development was achieved using 3,3',5,5'-Tetramethylbenzidine solution and the absorbance was recorded at a wavelength of 450 nm. A standard curve was interpolated to determine the amount of VEGF released from the hydrogel at each predetermined time point. Results were presented in terms of cumulative release of VEGF as a function of time.

### In vivo study

#### Animals and drug selection

Twenty-four female Sprague Dawley (SD) rats, 10–12 weeks old (Animal Resource Centre, Murdoch, Western Australia) weighing approximately 200 g were utilized in this study. Zoledronic acid monohydrate powder (Sigma-Aldrich, Castle Hill, NSW, Australia) was dissolved in sterile water (1 mg mL^−1^) and further diluted for administration. The protocol for this study were approved by the Animal Ethics Committee of Griffith University (DOH0414-AEC) and conducted in accordance with relevant guidelines and regulations. Furthermore, all experiments were conducted and reported in accordance with the recommendations in the ARRIVE guidelines.

#### Group assignment and drug administration

**Figure 9:**
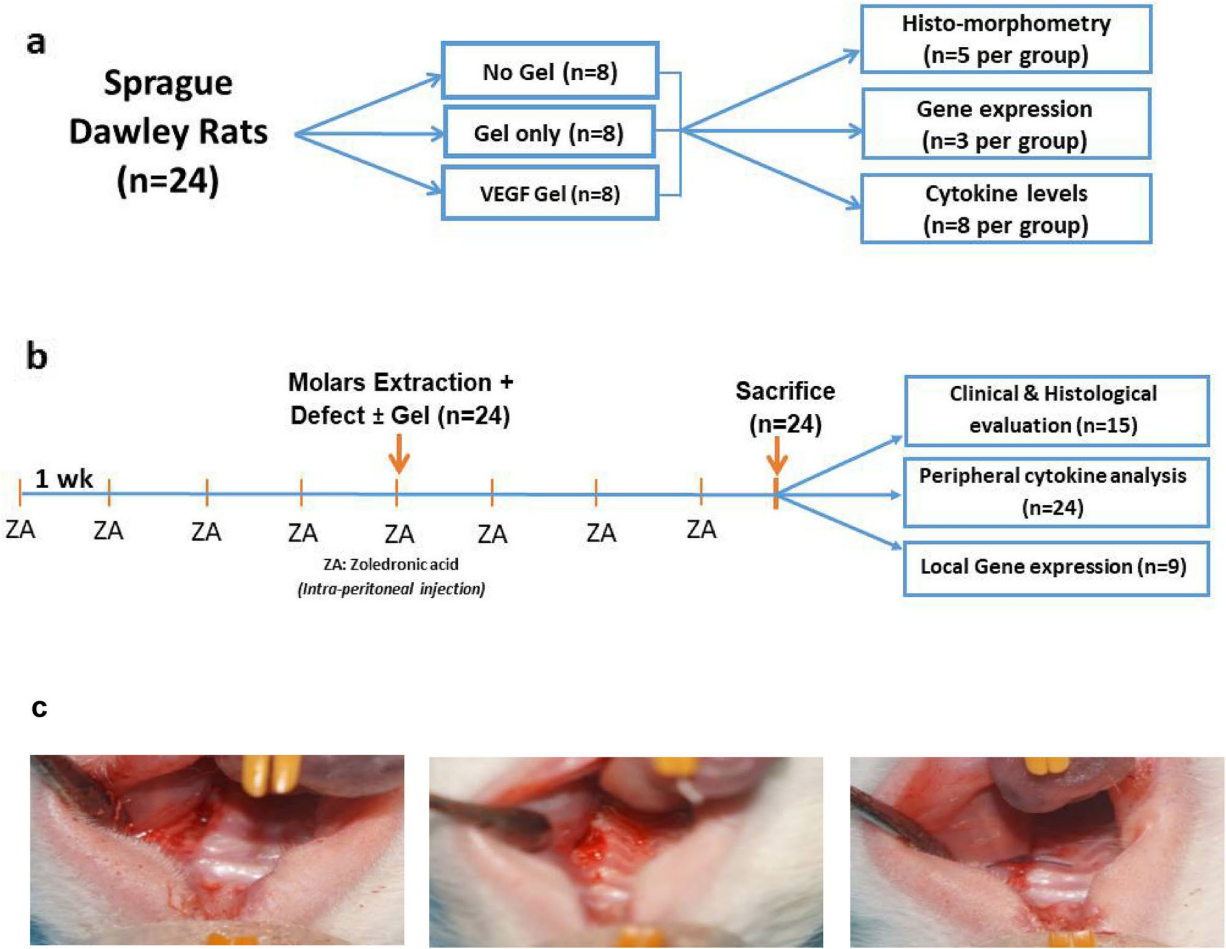
Group allocation of female Sprague Dawley rats for the in vivo study (**a**). Frequency of ZA administration, surgical protocol and timeline for the induction of MRONJ followed by treatment with or without VEGF-gel (**b**). Surgical procedure involving the extraction of maxillary molars, creation of defect and coronally advanced flap closure after gel placement is demonstrated in (**c**).

Intraperitoneal injection was chosen as the route of administration as this approach is known to induce MRONJ-like lesions in rodents^71–73^. In rodents, tissue distribution of bisphosphonate(s) is known to be independent of route administered and drug concentration in bone was noted to be essentially identical when medicaments were delivered either as intravenous, intraperitoneal or subcutaneous injection^74,75^. All rats were acclimatized for one week and the baseline body weight of each rat was recorded. Zoledronic acid (ZA) was administered once a week (198 µg per kg body weight) using a 23-gauge needle for the entire duration of the study. In our study, at week five of ZA administration, all rats were scheduled for surgery. The rats were randomly assigned to receive either the hydrogel with VEGF (VEGF-gel), the hydrogel alone (gel-alone), or no hydrogel (no-gel) after dental extraction (8 animals/group, Fig. 9).

#### Surgical protocol

All surgeries were performed under aseptic conditions to minimize the likelihood of infection. General anesthesia was administered via a nose cone using a Mediquip Isoflurane vaporizer (Attane, Bomac Animal Health Pty Ltd, Australia) set to 5% for induction then reduced to 1–3% for maintenance. The rats were then placed in a dorsal recumbency and the maxillary first and second molars (unilateral, randomly assigned) were extracted followed by the creation of a surgical defect (5 mm in diameter) within the extraction site using a rotary surgical bur with simultaneous saline irrigation to minimize thermal injury to the maxillary bone. Haemostasis was achieved via pressure on the wound prior to delivering the hydrogels (VEGF-gel at 200 ng mL^−1^ or gel-alone) into the prepared defect using a 1 mL syringe with an 18-gauge needle until the defect was filled with gel. To stabilize and prevent dislodgement of gel, primary closure was achieved to the maximum extent possible, utilizing a coronally advanced flap and resorbable simple interrupted sutures (Fig. 9c). All animals were provided with food and water ad libitum post-operatively and received a further weekly injection of ZA over the following four weeks of healing post-extraction.

#### Sample collection and gross morphological examination

Immediately prior to euthanasia, 3 ml of whole blood was collected by cardiac puncture for cytokine analysis^76^. Plasma was separated after blood clotting by centrifugation and stored at − 80 °C until the cytokine assay was performed. All the rats were euthanized 4 weeks after the surgery using carbon dioxide at 70% volume within the chamber and gross morphological examination was performed subsequently. The defect sites (n = 8 per group) were evaluated for signs of osteonecrosis including features of incomplete/delayed wound closure, bone exposure or frank necrotic changes. The grading of osteonecrosis was carried out as described by Ruggiero et al.; Grade 0 = healed mucosa and no apparent necrotic bone at the extraction site, 1 = no clinical evidence of necrotic bone but nonspecific findings at the extraction site, 2 = exposed/necrotic bone with no signs of infection, 3 = exposed/necrotic bone with signs of infection at the site of extraction, 4 = exposed and necrotic bone with infection and exposed and necrotic bone extending beyond the region of alveolar bone^77^.

Results were represented as the percentage of total sites with a particular grade of MRONJ within each group. Thereafter, the samples (n = 5) were retrieved and immediately fixed in a 4% paraformaldehyde solution overnight at room temperature prior to histological processing. Gene expression profile within the soft and hard tissue was also assessed. Fresh specimens (n = 3 per group) were dissected and the surrounding gingiva and the alveolar bone within the defect were carefully separated using a scalpel and immediately transferred into separate tubes containing Tri-reagent (Sigma-Aldrich, castle Hill, NSW, Australia).

#### Histomorphometric analysis

The maxillae were decalcified in 10% ethylenediaminetetraacetic acid (EDTA) solution (pH 7.2) with weekly changes of solution over 8 weeks. The samples were subsequently processed and embedded in paraffin wax allowing the sectioning along the parasagittal plane. Sections (4 µm thick) were obtained, stained with H&E, and three representative sections were selected per maxilla specifically to include buccal and mid-socket regions that was determined based on the morphology of the remaining molar in the maxillae. The slides were scanned using the Aperio Scanscope Digital Slide Scanner (Leica Biosystems Inc, Buffalo Grove, IL, USA) and histomorphometric analysis was performed. All quantification was performed by a single observer and repeated twice.

#### Quantification of osteonecrosis

Areas of osteonecrosis as evidenced histologically by regions of empty lacunae, were evaluated within the healing extraction sockets. Briefly, regions of > 500 μm^2^ were examined^78^ using the Aperio Imagescope software for bone sequestrations (of any size exhibiting empty lacunae) that is considered to be a hallmark of rodent MRONJ as reported by Biguetti et al. previously^61^. The annotation tool on the Imagescope software was used to mark the area of osteonecrosis (µm^2^), which was quantified and compared between the groups.

#### Quantification of inflammatory infiltrate

Intense leukocyte infiltration is typically found within areas of MRONJ in rodents^61^. Inflammatory component(s) around the sequestration on the histological sections were quantified in our study as described below. The total inflammatory infiltrate (µm^2^) was annotated using the pen tool in the Aperio Imagescope software which automatically quantified the area of annotation. Although inflammatory infiltrate does not usually possess a definite outline, all efforts were made to include only the region comprising dense cellular infiltrate (> 100 cells per 100 µm^2^) around the sequestrum to ensure standardized quantification.

#### Quantification of vascularity

Paraffin embedded sections were stained for the vascular markers von Willebrand factor (vWF) and CD105, a marker for neo-angiogenesis utilizing a simultaneous double immunofluorescence protocol. Briefly, de-waxed sections were affixed to adhesive slides (Menzel-Gläser Polysine) and treated with Proteinase K (Dako North Sydney, NSW Australia) and underwent antigen retrieval, followed by peroxide quenching by incubation in Hydrogen Peroxide (3% for 15 min). Non-specific antibody binding was inhibited by incubating the sections in BSA (1% in PBST) for 30 min followed by incubation with a mixture of the two primary antibodies, anti-vWF (1:100; ab6994, abcam Melbourne, VIC Australia) and anti-CD105 (1:50; ab11414, abcam Melbourne, VIC Australia) for one hour at 37 °C. Subsequently, sections were incubated with a mixture of two secondary antibodies with two different fluorochromes (DyLight® 650 for VEGF and FITC for anti-CD105) for one hour at room temperature in the dark followed by a nuclear stain (0.25 µg mL^−1^ DAPI in PBS for 2 min). The sections were imaged using a confocal microscope (Olympus FV1000 confocal laser imaging system, Melbourne, VIC Australia) and the quantification of each antigen was performed using threshold color calculations on the ImageJ software (ImageJ, U. S. National Institutes of Health, Bethesda, Maryland, USA, http://imagej.nih.gov/ij/).

#### Gene expression analysis

Real-time PCR was used to assess the gene expression of vascular (vWF, VEGF-R1, VEGF-R2, CD105), inflammatory (IL-1β & TNF-α) and bone (OCN, TRAP, ALP) markers in both soft (gingiva) and hard (bone) tissues from the defect site that were separated using blunt dissection. The samples were pulverized using a high speed rotary homogenizer after being transferred into Tri reagent (Sigma-Aldrich, castle Hill, NSW, Australia) and total RNA was extracted using standard protocols^79^.

Total RNA was quantified using a Nanodrop spectrophotometer (Thermo Scientific, Rockford, IL, USA). First-strand cDNA synthesis was carried out using 1 µg of RNA with oligo dT, 5X reaction buffer, MgCl_2_, dNTP mix, RNAse inhibitor and M-MLV Reverse Transcriptase (Promega, Madison, WI, USA), as per the manufacturer’s instructions. Real time PCR was performed using 2 µL cDNA, PCR master mix (KAPA SYBR FAST qPCR Kit, Kapa Biosystems, Boston, MA, USA) and forward and reverse primers (Integrated DNA Technologies, San Diego, CA, USA) on a Real Time PCR system (IQ5, BioRad Laboratories, Hercules, CA, USA). The specificity of the primers (Table 1) was determined by melt curve analysis at the end of each run. The PCR cycling conditions used were 3 min at 95 °C for enzyme activation, 40 cycles of 3 s at 95 °C, 20 s at 52–58 °C (depending upon the primer annealing temperatures) and 2 s at 72 °C. Each real-time PCR was carried out in triplicate and the threshold cycle values were averaged. Calculation of relative gene expression was carried out using the ΔΔCt method. The results were normalized against the housekeeping gene glyceraldehyde-3-phosphate dehydrogenase (GAPDH).

**Table 1:** Primer sequences, utilized for gene expression analysis.

Gene	Reference sequence	Primers	Product length
vWF	NM_053889.1	FP:5′-CCGGAAGCGACCCTCAGA-3′ RP: 5′-CGGTCAATTTTGCCAAAGATCT-3′	123
VEGF-R1	NM_019306.2	FP: 5′-CGTGGTCAAACTCTTGTCCTCAAC-3′ RP: 5′-CGGTCAATTTTGCCAAAGATCT-3′	113
VEGF-R2	NM_013062.1	FP: 5′-TCAATGTGGTGAACCTGCTGG-3′ RP: 5′-TTCTCTTGCCCCGTAAGTAAGTTG -3′	111
CD105	NM_001010968.2	FP: 5′-GGCTATGCCATGCTGCTGGTGG -3′ RP: 5′-GGTACATCTACTCTCACAC-3′	148
IL-1β	NM_031512.2	FP: 5′-CACCTCTCAAGCAGAGCACAG-3′ RP: 5′-GGGTTCCATGGTGAAGTCAAC-3′	79
TNF-α	NM_031512.2	FP:5′-TCTGCTTGGTGGTTTGCTACGAC-3′ RP:5′-TCTGCTTGGTGGTTTGCTACGAC-3′	111
OCN	NM_013414.1	FP:5′-GGTGCAAAGCCCAGCGACTCT-3′ RP:5′-GGAAGCCAATGTGGTCCGCTA-3′	199
TRAP	NM_019144.2	FP:5′-CGCCAGAACCGTGCAGA-3′ RP:5′-TCAGGCTGCTGGCTGAC-3′	357
ALP	NM_013059.1	FP:5′-TGCAGGATCGGAACGTCAAT-3′ RP: 5′-GAGTTGGTAAGGCAGGGTCC-3′	143
GAPDH	NM_001025109.1	FP: 5′-TGTGTCCGTCGTGGATCTGA-3′ RP: 5′-TTGCTGTTGAAGTCGCAGGAG-3	150

#### Peripheral blood cytokine assay

A multiplex cytokine assay (Bio-Plex, Bio-Rad, Gladeville, NSW) was used to quantify the levels of six pro- and anti-inflammatory cytokines: Interleukin (IL) 1α & 1β, Tumor Necrosis Factor-α (TNF-α), RANTES, Monocyte chemotactic protein 1 (MCP-1), Macrophage inflammatory protein 1 (MIP-1) along with the vascular marker, VEGF, in peripheral circulation as per the manufacturer’s instructions.

### Statistical analysis

Results are expressed as mean ± standard error and a one-way ANOVA with post hoc testing (Tukey) was used to analyze the data. GraphPad 8.4 (GraphPad Software, CA, USA) for windows was used for analysis and p < 0.05 was considered to be statistically significant.
